# Retraction Note: TRIM25 blockade by RNA interference inhibited migration and invasion of gastric cancer cells through TGF-β signaling

**DOI:** 10.1038/s41598-023-32830-y

**Published:** 2023-04-05

**Authors:** Zhenya Zhu, Yong Wang, Chunhui Zhang, Shiyong Yu, Qi Zhu, Kun Hou, Bo Yan

**Affiliations:** 1 Department of General Surgery, Shanghai Pudong District People’s Hospital, Shanghai, 201299 China; 2 Department of General Surgery, Punan Hospital, Pudong New District, Shanghai, 200125 China

Retraction of: *Scientific Reports* 10.1038/srep19070, published online 12 January 2016

The Editors have retracted this Article.

After publication it was brought to the Editors’ attention that the Article contains multiple instances of overlapping images both within figures and with figures in other publications reporting different experiments. The Editors therefore no longer have confidence in the reliability of the data published in this Article.

The Editors were not able to obtain a current email address for any of the authors.

